# Potential Added Value of PET/CT Radiomics for Survival Prognostication beyond AJCC 8th Edition Staging in Oropharyngeal Squamous Cell Carcinoma

**DOI:** 10.3390/cancers12071778

**Published:** 2020-07-03

**Authors:** Stefan P. Haider, Tal Zeevi, Philipp Baumeister, Christoph Reichel, Kariem Sharaf, Reza Forghani, Benjamin H. Kann, Benjamin L. Judson, Manju L. Prasad, Barbara Burtness, Amit Mahajan, Seyedmehdi Payabvash

**Affiliations:** 1 Section of Neuroradiology, Department of Radiology and Biomedical Imaging, Yale School of Medicine, 789 Howard Ave, New Haven, CT 06519, USA; stefan.haider@yale.edu (S.P.H.); amit.mahajan@yale.edu (A.M.); 2 Department of Otorhinolaryngology, University Hospital of Ludwig Maximilians Universität München, Marchioninistrasse 15, 81377 Munich, Germany; philipp.baumeister@med.uni-muenchen.de (P.B.); christoph.reichel@med.uni-muenchen.de (C.R.); kariem.sharaf@med.uni-muenchen.de (K.S.); 3 Center for Translational Imaging Analysis and Machine Learning, Department of Radiology and Biomedical Imaging, Yale School of Medicine, 333 Cedar Street, New Haven, CT 06510, USA; tal.zeevi@yale.edu; 4 Department of Diagnostic Radiology and Augmented Intelligence & Precision Health Laboratory, McGill University Health Centre & Research Institute, 1650 Cedar Avenue, Montreal, QC H3G 1A4, Canada; reza.forghani@mcgill.ca; 5 Department of Radiation Oncology, Dana-Farber Cancer Institute, Harvard Medical School, 450 Brookline Avenue, Boston, MA 02215, USA; benjamin_kann@dfci.harvard.edu; 6 Division of Otolaryngology, Department of Surgery, Yale School of Medicine, 330 Cedar Street, New Haven, CT 06520, USA; benjamin.judson@yale.edu; 7 Department of Pathology, Yale School of Medicine, 310 Cedar Street, New Haven, CT 06520, USA; manju.prasad@yale.edu; 8 Section of Medical Oncology, Department of Internal Medicine, Yale School of Medicine, 25 York Street, New Haven, CT 06520, USA; barbara.burtness@yale.edu

**Keywords:** radiomics, oropharyngeal squamous cell carcinoma, PET/CT, quantitative imaging, HPV, imaging biomarker, survival analysis, risk stratification, head and neck cancer

## Abstract

Accurate risk-stratification can facilitate precision therapy in oropharyngeal squamous cell carcinoma (OPSCC). We explored the potential added value of baseline positron emission tomography (PET)/computed tomography (CT) radiomic features for prognostication and risk stratification of OPSCC beyond the American Joint Committee on Cancer (AJCC) 8th edition staging scheme. Using institutional and publicly available datasets, we included OPSCC patients with known human papillomavirus (HPV) status, without baseline distant metastasis and treated with curative intent. We extracted 1037 PET and 1037 CT radiomic features quantifying lesion shape, imaging intensity, and texture patterns from primary tumors and metastatic cervical lymph nodes. Utilizing random forest algorithms, we devised novel machine-learning models for OPSCC progression-free survival (PFS) and overall survival (OS) using “radiomics” features, “AJCC” variables, and the “combined” set as input. We designed both single- (PET or CT) and combined-modality (PET/CT) models. Harrell’s C-index quantified survival model performance; risk stratification was evaluated in Kaplan–Meier analysis. A total of 311 patients were included. In HPV-associated OPSCC, the best “radiomics” model achieved an average C-index ± standard deviation of 0.62 ± 0.05 (*p* = 0.02) for PFS prediction, compared to 0.54 ± 0.06 (*p* = 0.32) utilizing “AJCC” variables. Radiomics-based risk-stratification of HPV-associated OPSCC was significant for PFS and OS. Similar trends were observed in HPV-negative OPSCC. In conclusion, radiomics imaging features extracted from pre-treatment PET/CT may provide complimentary information to the current AJCC staging scheme for survival prognostication and risk-stratification of HPV-associated OPSCC.

## 1. Introduction

Over past decades, the incidence of oropharyngeal squamous cell carcinomas (OPSCC) has continuously increased, which is attributed to a marked rise in prevalence of sustained high-risk human papillomavirus (HPV)-infection in the oropharynx [1,2,3,4]. Despite arising from the same pharyngeal site, HPV-associated and HPV-negative OPSCC are considered separate cancer entities with diverging demographic, biologic, and, most notably, prognostic characteristics. HPV-positive cancers are associated with longer overall survival (OS), progression-free survival (PFS), and more favorable treatment response as compared to the HPV-negative form [5,6,7,8]. Consequently, the 8th edition of the American Joint Committee on Cancer (AJCC)/Union for International Cancer Control (UICC) staging manuals adopted separate staging schemes for survival risk-stratification and prognostication of HPV-associated and HPV-negative OPSCC [9,10,11,12].

Advancements in high-throughput computing and machine-learning led to emergence of the “-omics” concept, referring to collective characterization and quantification of pools of biologic information, such as genomics, proteomics, or metabolomics. Radiomics refers to automated extraction of high-dimensional, quantitative descriptor (“feature”) sets from medical images for various applications, including survival modelling, treatment guidance, and biomarker design [13,14,15,16,17]. Such features correlate with clinical outcome and convey medically meaningful information describing tumor heterogeneity, microenvironment, pathophysiology, and mutational burden [13,18,19]. While prior studies demonstrated prognostic value of radiomics biomarkers in head and neck cancers [15,16,20,21,22,23,24,25,26,27,28], none have incorporated or compared the AJCC 8th edition staging scheme in OPSCC survival modelling and stratification. In this study, we explored the potential added value of radiomics biomarkers in prognostication of PFS and OS—beyond the AJCC staging scheme—in a multi-institutional cohort. 

[18F]Fluorodeoxyglucose positron emission tomography (PET) and computed tomography (CT) are a mainstay of OPSCC staging, treatment planning, and surveillance. We applied machine-learning algorithms to devise prognostic radiomics biomarkers for OPSCC using baseline PET and/or CT scans from a multi-institutional cohort. Then, we compared the radiomic biomarkers’ performance with AJCC staging for prognostication and risk-stratification of PFS and OS in HPV-associated and HPV-negative subgroups. 

## 2. Results

### 2.1. Cohort Characteristics

For the PFS study arm, 311 OPSCC patients (235 HPV-associated and 76 HPV-negative) met inclusion criteria; 94 (30.2%) experienced events. For the OS study arms, 306 OPSCC patients (233 HPV-associated and 73 HPV-negative) met inclusion criteria; 58 (19.0%) died. The AJCC staging, demographics, treatment, and imaging characteristics are summarized in Table 1, and are separately reported for HPV subgroups in Tables S1 and S2. 

### 2.2. Survival Model Performance

**Figure 1:**
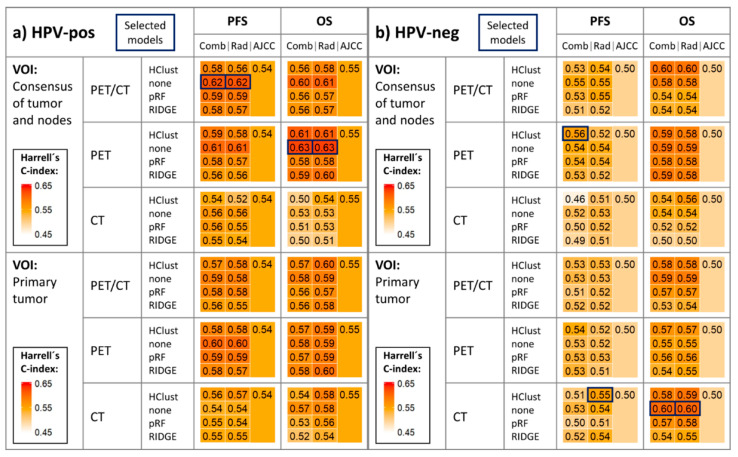
Heatmap depicting mean Harrell’s C-index in validation folds across 33 repeats of threefold stratified cross-validation in the (**a**) HPV-associated and (**b**) HPV-negative cohorts. Models selected for further evaluation are highlighted in the figure. All 24 methodological approaches to generate optimized radiomics signatures for radiomics and combined models were applied in each study arm (OS and PFS): 4 dimensionality reduction techniques (HClust, none, pRF, RIDGE) × 2 volume of interest (VOI) sources × 3 imaging modalities. The corresponding AJCC models are also reported. Detailed mean Harrell’s C-index ± standard deviations are reported in Figure S1. AJCC = AJCC model; Comb = combined model; HClust = hierarchical clustering; none = no dimensionality reduction applied; OS = overall survival; PFS = progression-free survival; pRF = Pearson correlation-based redundancy reduction with random survival forest variable importance; Rad = radiomics model; RIDGE = Cox regression with RIDGE regularization adapted for feature selection.

OPSCC random survival forest (RSF) models combining radiomics features and AJCC staging (T-, N-, and overall-stage) yielded higher averaged Harrell´s C-index scores than AJCC models in the PFS and OS study arms for both HPV-associated and HPV-negative cohorts in the majority of permutations (Figure 1 and Figure S1). Similarly, survival models relying on radiomics predictors alone outperformed AJCC baseline models in the majority of permutations (Figure 1 and Figure S1). 

In the HPV-associated subgroup, the best PFS radiomics model (using PET/CT consensus volume of interest (VOI) radiomics features) achieved an average C-index ± SD of 0.62 ± 0.05 (*p* = 0.02) compared to 0.54 ± 0.06 (*p* = 0.32) from AJCC staging; and the best OS radiomics model (using PET consensus VOI radiomics features) yielded 0.63 ± 0.08 (*p* = 0.06) compared to 0.55 ± 0.08 (*p* = 0.34) from AJCC staging (Figure 1 and Figure S1). 

In the HPV-negative subgroup, the best PFS radiomics model (using CT primary tumor radiomics features, and hierarchical clustering feature reduction) achieved an average C-index ± SD of 0.55 ± 0.07 (*p* = 0.25) compared to 0.50 ± 0.06 (*p* = 0.51) from AJCC staging, and the best OS radiomics model (using CT primary tumor radiomics features) yielded 0.60 ± 0.09 (*p* = 0.17) compared to 0.50 ± 0.08 (*p* = 0.53) from AJCC staging. Combined models utilizing radiomics features and AJCC variables yielded similar performance (Figure 1 and Figure S1). 

### 2.3. Time-Dependent Survival Model Evaluation

**Figure 2:**
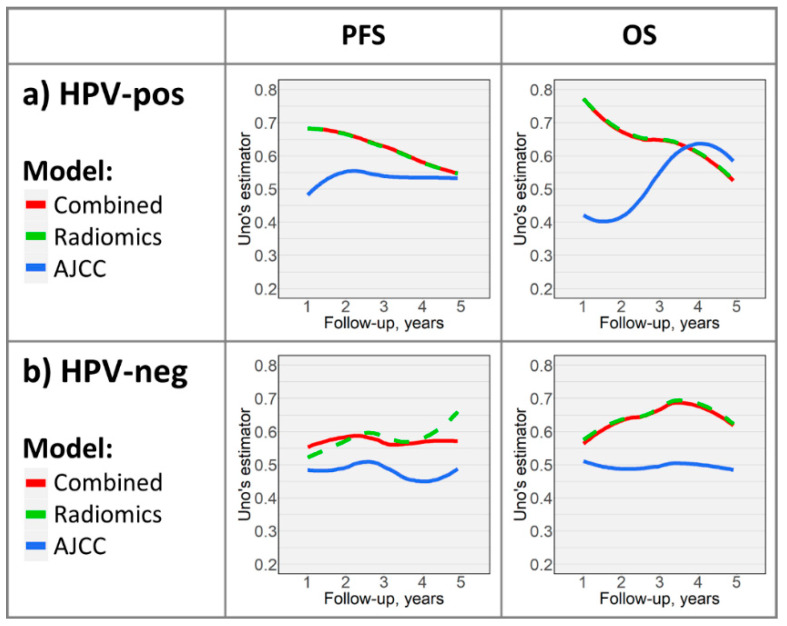
Time-dependent performance curves depicting select models’ (highlighted in Figure 1) prognostic abilities throughout follow-up in the (**a**) HPV-associated and (**b**) HPV-negative cohorts. For comparison, corresponding AJCC models’ curves are depicted.

Models selected for further evaluation are highlighted in Figure 1. Performance curves (Figure 2) demonstrate time-dependent performance of PFS and OS models. The radiomics and combined PFS models had superior prognostic accuracy compared to the AJCC model throughout 5 years of follow-up in HPV-associated and HPV-negative cohorts. In the OS study arm, performance curves revealed superiority of radiomics-based models throughout follow-up in the HPV-negative cohort; however, while the radiomics and combined models outperformed the AJCC model in the first ≈3.5 years of follow-up in the HPV-associated cohort, the AJCC model was slightly superior thereafter. 

### 2.4. Kaplan–Meier Analysis

**Figure 3:**
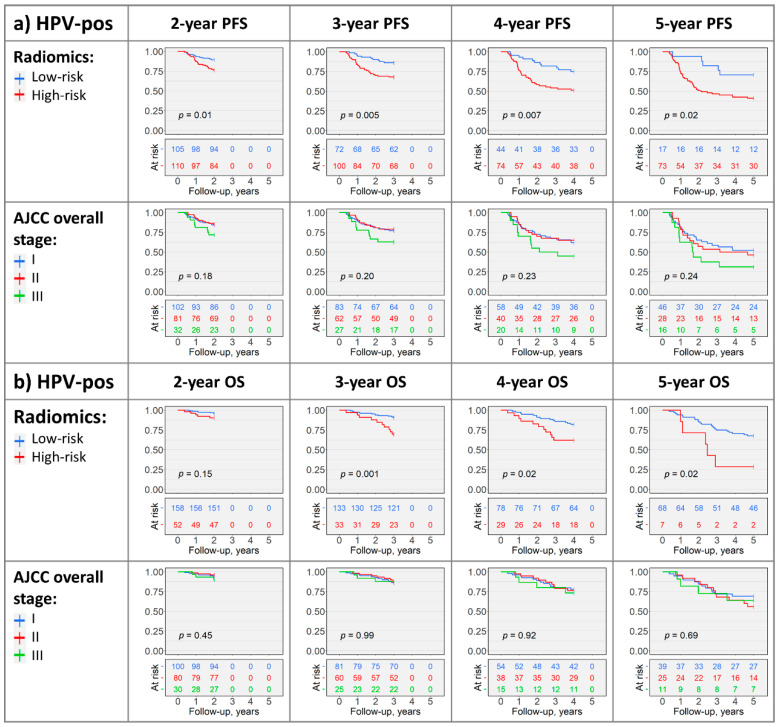

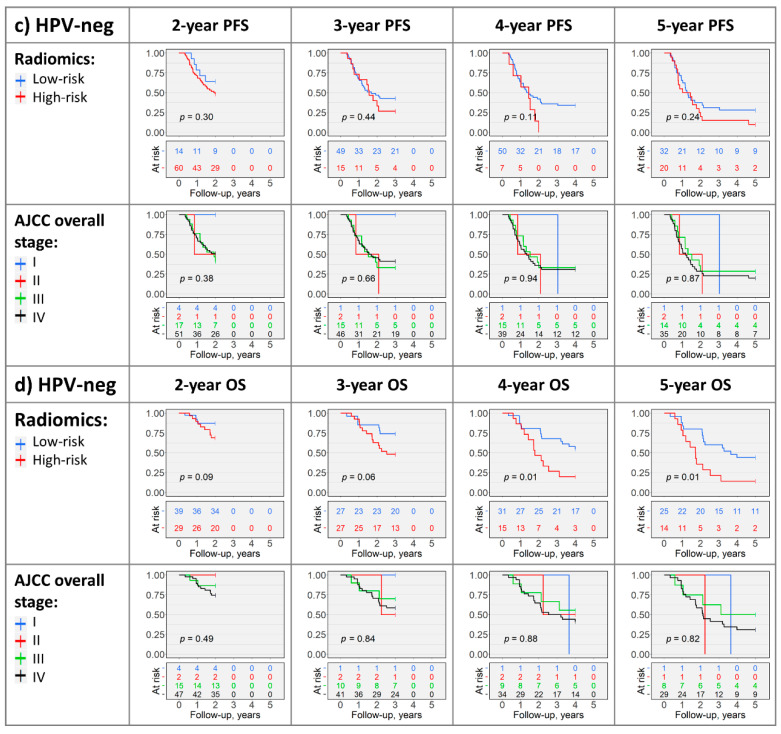
Kaplan–Meier plots with log-rank test *p*-values depicting radiomics- and AJCC overall stage risk-stratification in HPV-associated (**a**,**b**) and HPV-negative (**c**,**d**) cohorts in the OS and PFS study arms. The AJCC T- and N-stage Kaplan–Meier plots, and plots with grouped AJCC variables are included in Figure S2.

Radiomics-based PFS risk stratification was significant in the HPV-associated cohort (for 2-, 3-, 4-, and 5-year PFS: *p* = 0.01, *p* = 0.005, *p* = 0.007, and *p* = 0.02, respectively), whereas AJCC staging was not significant (*p* > 0.05; Figure 3a and Figure S2a). Pooling N-stages (N0 + N1 vs. N2 + N3) yielded significant 2-year and 3-year PFS stratification; however, radiomics risk groups had more balanced subject counts (Figure S2a). Neither radiomics nor AJCC staging could significantly differentiate PFS in the HPV-negative cohort (*p* > 0.05, Figure 3c and Figure S2c). 

The 3-, 4-, and 5-year radiomics-based OS stratifications were significant in the HPV-associated cohort (*p* = 0.001, *p* = 0.02, and *p* = 0.02, respectively), while the 2-year OS *p*-value approached significance (*p* = 0.15; Figure 3b). Similarly, 4- and 5-year radiomics risk groups had significantly different OS in the HPV-negative cohort (both *p* = 0.01), while the 2- and 3-year OS *p*-values approached significance (*p* = 0.09 and *p* = 0.06, respectively; Figure 3d). AJCC staging did not achieve significant OS stratification (*p* > 0.05; Figure 3b,d and Figure S2b,d). 

## 3. Discussion

Currently, pre-treatment imaging of head and neck cancers serves the purpose of evaluating primary tumor dimensions, anatomical extent, involvement of regional lymph nodes, and detecting distant metastases, which constitute main components of AJCC/UICC staging. However, our results suggest quantitative imaging biodata reflecting tissue density, texture patterns, lesion shape, and metabolic activity of primary tumors and metastatic cervical nodes may encode valuable information pertaining to tumor behavior with potential prognostic relevance. In both HPV-associated and HPV-negative OPSCC, we observed trends suggesting radiomic analysis may provide complementary value for prognostication and risk-stratification beyond AJCC staging. Statistical significance was consistently attained for PFS survival prognostication and risk-stratification in HPV-associated OPSCC. Additionally, radiomics-based OS risk-stratification outperformed AJCC staging variables in HPV-associated OPSCC, with similar trends in HPV-negative patients. Notably, models utilizing PET radiomics or combined PET and CT feature sets predominantly outperformed CT-based survival prognostication in the HPV-associated subgroup; additionally, consensus VOI models utilizing radiomics information from both primary tumor and metastatic nodes were usually superior. 

Our methodology may be applied in future larger cohorts to generate uniformly applicable and objective imaging biomarkers for prognostic risk-stratification of OPSCC. Additionally, our approach enables inclusion of additional prognostic variables into PFS and OS models for risk-stratification of head/neck cancer subgroups [29]. 

To enhance generalizability and model robustness against heterogeneity in imaging and reconstruction protocols and scanning equipment, we acquired a multi-institutional dataset provided by cancer centers in the United States and Canada. Overall, AJCC models had modest prognostic accuracy in HPV-positive and HPV-negative subsets, achieving an averaged C-index ± SD of up to 0.55 ± 0.08 (*p* = 0.34, OS analysis of HPV-positive patients), which is likely attributable to low event rates and relatively small cohort sizes. On the other hand, in the HPV-associated subgroup, a PET/CT radiomics model using the full set of consensus VOI features for PFS prognostication produced an averaged C-index ± SD of 0.62 ± 0.05 (*p* = 0.02), and a PET model using consensus features for OS prediction achieved 0.63 ± 0.08 (*p* = 0.06). We observed similar trends in HPV-negative OPSCC, despite using a smaller cohort. 

To illustrate models’ prognostic abilities throughout the follow-up period, we plotted performance curves (Figure 2). Findings from heatmaps are again reflected, with radiomics or combined models predominantly outperforming AJCC models. The differences between models were more notable in early years of follow-up, which could be related to data sparsity in later years of follow-up. It is likely feasible to train machine-learning models with improved long-term prognostication using larger cohorts with longer follow-up. 

Most prior OPSCC radiomics studies relied on generalizations of linear models to examine radiomics features and predict survival [22,23,24,25]. We applied a random forest machine-learning algorithm specifically designed to handle right-censored survival data (“random survival forest”) [30,31,32], with proven superiority in utilizing the full prognostic capability of radiomics data [28]. Decision tree growing—which is repeatedly performed in random forest training—resembles decision-making that physicians may apply in clinical practice—with multiple variables present, the algorithms may first select the most prognostic one (e.g., HPV-status) to stratify cases. Thereafter, further variables (e.g., AJCC-staging, radiomics feature) are incorporated in growing decision trees to sub-stratify patients and refine survival prognostication [30]. 

Moreover, radiomics-based stratification generated high-risk and low-risk groups with significantly different PFS and OS in HPV-associated OPSCC for the 3-, 4-, and 5-year follow-up endpoints (Figure 3). In comparison, AJCC 8th edition overall-, T-, and N-staging exhibited modest abilities in risk-stratification (Figure 3 and Figure S2), suggesting complementary value of radiomic features for OPSCC risk-stratification in addition to HPV-status and AJCC 8th edition staging. 

It should be noted that C-indices reported for AJCC models in our study were averaged across validation folds in repeated cross-validation analysis utilizing overall stage and T-/N-stage as prognostic variables, which is methodologically different from some prior studies [33,34,35]. Dissimilarities in analysis methodology, sample size, length of follow-up, and numbers of events may have contributed to the differences between AJCC model C-indices in our study and prior reports.

Despite using a multi-institutional cohort, the sample size and length of follow-up might not suffice for training radiomics models for long-term prognostication. Our study was also limited by its lack of fully independent validation in external cohorts and adjustment for other OPSCC outcome predictors. Regional metastatic spread for segmentation was determined by expert read of PET/CT scans, but without tissue sampling from all nodes. HPV-status was ascertained through following institutional standards in The Cancer Imaging Archive (TCIA) cohorts with a heterogenous array of testing methods. 

## 4. Materials and Methods 

### 4.1. Data Acquisition

We retrospectively acquired clinical and imaging data from (1) Yale’s Smilow Hospital cancer registry from 2009–2019, and (2) two publicly available TCIA collections [36]: the “Head-Neck-PET-CT” collection from four Canadian institutions (“Canadian” cohort) [37,38] and the “Data from Head and Neck Cancer CT Atlas” collection from MD Anderson Cancer Center (“MD Anderson” cohort) [39,40]. Institutional review board approval was obtained from the Yale University ethics committee (IRB protocol #2000024295) and informed consent was waived, given the retrospective study design. TCIA datasets are de-identified and providing entities ensure ethical compliance. 

Patients with (1) histopathologically confirmed OPSCC, (2) known HPV-status, (3) pre-treatment PET and non-contrast CT scans, and (4) complete follow-up information were included. We excluded patients (1) presenting with distant metastases upon initial staging, (2) receiving palliative therapy and/or denying treatment, (3) recurrent OPSCC at presentation, (4) with CT artifacts affecting >50% of the primary gross tumor volume on visual evaluation [41], and (5) with uneventful follow-up <18 months. Biopsies or cytologic sampling prior imaging were permissible.

Patients from Yale were regularly followed up for cancer surveillance with physical examinations, endoscopy, and imaging; additional tissue sampling was performed at oncologists’ discretion. Disease progression or recurrence was ascertained by biopsies or unequivocal imaging evidence; the latter was confirmed by additional tissue sampling or documented response to anticancer therapy. For TCIA cohorts, annotations provided within the datasets were utilized to determine study endpoints. HPV association was determined by high-risk HPV-specific [42] testing and/or p16-immunohistochemistry, and “Yale” test results were interpreted following the Guideline from the College of American Pathologists [42]. An overall HPV status is provided in TCIA for the “Canadian” cohort, reflecting institutional testing and interpretation, and a high-risk HPV in situ hybridization status was available for the “MD Anderson” dataset. PET/CT imaging and reconstruction were performed at the source institutions utilizing standard clinical protocols. 

### 4.2. Lesion Segmentation

**Figure 4:**
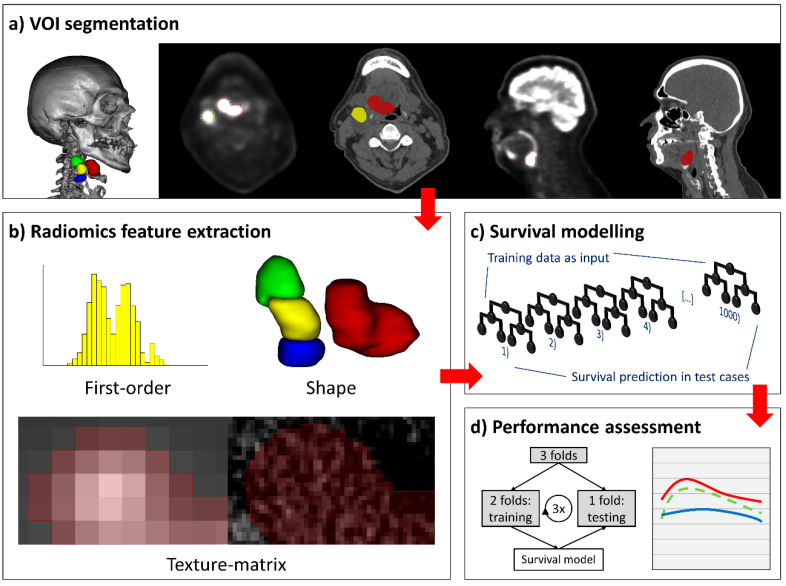
Segmentation, radiomics feature extraction, and survival modelling pipeline. (**a**) Positron emission tomography (PET)-guided manual segmentation of the primary tumor and individual metastatic cervical lymph nodes on PET and computed tomography (CT) axial slices, sagittal images, and a 3D-renderered image are provided for spatial awareness. (**b**) Extraction of first-order, shape, and texture matrix features yielded 1037 radiomics features per imaging modality and per lesion. (**c**) Random forest machine-learning models with 1000 decision trees were applied for survival prediction and risk-stratification. (**d**) Model performance was assessed in threefold cross-validation (left), wherein all subjects were assigned to three folds by stratified random split; the models were trained on two folds, and one fold was used for model validation. Model performance was visualized in performance curves (right).

To facilitate radiomics feature extraction, we defined separate PET and CT VOI for primary tumors and individual metastatic cervical lymph nodes. Each lesion was manually contoured (“segmented”) on PET axial slices, and segmentations were transferred to the co-registered CT and adapted to exclude uninvolved bone, air, and preserved fat planes. CT axial images with streak artifacts affecting the VOI were excluded from analysis on the basis of visual assessment, and lymph nodes with artifacts in >50% of the VOI were entirely disregarded [41]. Segmentations were verified and adjusted by experienced neuroradiologists, who additionally performed cancer staging according to the 8th edition AJCC Manual [9]. We utilized 3D-Slicer version 4.10.1 for image review and VOI segmentation [43,44]. Figure 4 summarizes the segmentation and feature extraction pipeline.

### 4.3. Radiomics Feature Extraction 

An automated image pre-processing pipeline facilitated homogenized radiomics analysis [19]. As detailed in the supplementary methods, we performed PET grey scale normalization, PET/CT voxel size homogenization, CT re-segmentation, generation of 10 derivative images per original scan, and grey scale discretization prior to radiomics feature extraction. 

Subsequently, we extracted 1037 PET and 1037 CT radiomics features per primary tumor or lymph node: 18 first-order and 75 texture-matrix features from each lesion’s representation in the original and derived PET and CT images, and 14 volumetric shape features from the original series (Table S3 includes a comprehensive list of features). We customized a Pyradiomics version 2.1.2 pipeline for image pre-procession and feature extraction [45,46]. 

Given the variable robustness of individual radiomics features to inter- and intra-observer segmentation inconsistencies [47,48,49,50,51,52], we determined feature stability, retaining only stable features for analysis; the methodology and results are reported in the supplementary methods and Table S4 [19]. 

### 4.4. Survival Study Arms and Cohorts

Survival was defined as the time interval from OPSCC diagnosis to the first event in a study arm, with censoring applied at loss of follow-up. Events in the PFS study arm were defined as locoregional recurrence or progression, distant metastasis, or death from any cause, and events in the OS study arm were deaths from any cause. Patients with uneventful follow-up <18 months were excluded from each respective study arm. This approach allows training the prognostic algorithm on survival data with adequate event-density in early follow-up, avoiding event sparsity-related performance deterioration. Survival analysis in each study arm was separately performed for the HPV-associated and HPV-negative study cohorts. 

### 4.5. Survival Modelling

For each study arm, we generated survival models using (1) clinical “AJCC” staging, i.e., overall stage, T-stage, and N-stage; (2) “radiomics” signatures; and (3) “combined” models using AJCC staging and radiomics signatures. Survival models were fitted on the combined dataset including all subjects and were evaluated in HPV-associated and HPV-negative study cohorts. AJCC features were concatenated with HPV status and were included as categorical variables, including overall stage with seven levels (I–IV and I–III in HPV-negative and HPV-associated cancers, respectively), and T- and N- stage with eight levels each (T1-T4 and N0-N3 in HPV-negative and HPV-associated cancers, respectively). Since patients with distant metastasis were excluded from analysis, no HPV-associated stage IV patients were included. 

We compared several approaches to generate optimized radiomics signatures for radiomics and combined models. Three feature dimensionality reduction techniques were compared to the prognostic performance of the full feature set (details in the supplementary methods; abbreviations in Figure 1). Feature sets were derived from two VOI sources of radiomics input: (a) the primary tumor lesion, and (b) consensus of the primary tumor and all metastatic cervical nodes (i.e., “virtual” consensus VOI as described by Yu et al. [53]). Feature sets from three imaging modalities (PET, CT, PET and CT) were utilized for model development. All 24 methodological combinations (4 dimensionality reduction techniques × 2 VOI sources × 3 imaging modalities) were applied in each study arm. 

R version 3.6.0 was utilized for statistical analysis [54]. Using the predictor sets (AJCC, radiomics and combined) described above, we trained and evaluated random survival forest (RSF) [30] models using the “ranger” package (version 0.12.1) [32] configured to grow 1000 trees per forest using a C-index split rule [31]. Other parameters were set according to default package recommendations. 

### 4.6. Cross-Validation and Performance Evaluation of Survival Models

We devised a framework applying 33 repeats of threefold stratified cross-validation to assess prognostic model performance, with the event/nonevent groups, HPV-status groups, and time to event/censoring as strata. In each cross-validation iteration, consensus VOI generation (if applicable), radiomics feature standardization, dimensionality reduction, and RSF training were consecutively performed on the training folds, and RSF performance was evaluated in the validation fold. This strategy yields accurate estimates of models’ prognostic capability in new cohorts, as “information leakage” between folds is rigorously avoided. 

Harrell’s C-index [31,55,56] quantified model performance in validation folds, and each model’s score was averaged across all 99 cross-validation permutations. We selected the radiomics and combined models yielding the highest average C-index per each combination of study cohort (HPV-associated and HPV-negative) and study arm (PFS and OS) for further evaluation in those respective datasets. 

A corrected paired *t*-test (“corrected repeated k-fold cv test” [57,58]) was applied to compare select models’ C-index distribution across validation folds against random predictions (i.e., the same fitted models applied in validation folds with randomly resampled survival outcome). 

Uno’s estimator of cumulative/dynamic area under the curve (AUC) for right-censored survival data [59,60] was computed in each validation fold to track model performance throughout follow-up, and was averaged across 33 cross-validation repeats. The resulting time-dependent performance curves were plotted for 5 years of follow-up. The radiomics data of selected models were utilized in risk-stratification analysis. 

We used the R “Hmisc” package [61] for C-index calculation, the “survAUC” package [62] to compute Uno’s AUC estimator, and the “geom_smooth” function implemented in “ggplot2” (version 3.2.1) [63] to apply “LOESS” smoothing [64] on performance curves. 

### 4.7. Risk-Stratification and Kaplan–Meier Analysis

To investigate the potentials of quantitative imaging for risk-stratification in HPV-associated and HPV-negative OPSCC, we utilized radiomics features for binary classification (high-risk vs. low-risk) and subsequently subjected cohorts to Kaplan–Meier analysis. For risk-stratification, we used random classification forest (RCF) models (“ranger” package version 0.12.1) [32] configured to grow 1000 trees per forest with the remaining parameters in default setting. The framework applying 33 repeats of threefold stratified cross-validation was adapted, with the event/nonevent groups as strata. Each patient’s RCF output (probability of experiencing an event) was averaged across validation folds to generate risk scores. A cutoff was selected by maximizing Youden’s index in receiver operating characteristic analysis, and patients with risk scores greater than the cutoff were assigned to the “radiomics” high-risk group. 

All classified patients were subjected to Kaplan–Meier analysis with their radiomics risk group, and a log-rank test ascertained statistical significance defined as *p* < 0.05. For comparison, AJCC overall stage groups, T-stage, and N-stage were applied for risk-stratification. 

The radiomics-only datasets of survival models selected for further evaluation were used as RCF input without feature reduction applied, and risk-stratification models were trained and evaluated separately in each study cohort and study arm. To label subjects for Kaplan–Meier analysis, cutoffs corresponding to 2, 3, 4, and 5 years of follow-up were used; patients experiencing events before a given cutoff were labeled as positive instances, subjects lost-to-follow-up before a cutoff were excluded, and all remaining patients were labelled negative and censored at the cutoff. RCF models were trained for each cutoff, and separate Kaplan–Meier plots were generated using radiomics risk groups and AJCC variables for risk-stratification. This approach allows supplying “dense” survival data to RCF algorithms (i.e., no censoring) while enabling accurate comparison with AJCC stratification. 

## 5. Conclusions

Pre-treatment PET/CT radiomics biomarkers may provide complementary prognostic value for OPSCC beyond AJCC/UICC 8th edition staging via systematic quantification of tissue density, texture patterns, lesion geometry, and metabolic properties. Our results suggest an added value of radiomics biomarkers for survival prognostication and risk-stratification in HPV-associated OPSCC, with similar trends in HPV-negative cancers. Pending careful development and rigorous validation in larger multi-institutional/multi-national datasets, radiomics markers may improve prognostication and risk-stratification in a clinical setting and pave the road for personalized treatment and targeted therapy.

## Figures and Tables

**Table 1:** Patients’ Characteristics.

Survival Endpoint	Progression-Free Survival	Overall Survival
**Number of patients**^1^—*n*	311	306
**Included lymph nodes**—*n*	475	462
**Events**—*n* (%)	94 (30.2%)	58 (19.0%)
**Follow-up (days)**—median (IQR)	1170 (798–1645)	1197 (818–1656)
**Data source**—*n* (%)		
Yale	201 (64.6%)	200 (65.4%)
TCIA	110 (35.4%)	106 (34.6%)
**Sex**—*n* (%)		
Male	253 (81.4%)	249 (81.4%)
Female	58 (18.6%)	57 (18.6%)
**Age (years)**—mean (SD)	60.61 (9.24)	60.60 (9.28)
**HPV status**^2^—*n* (%)		
Positive	235 (75.6%)	233 (76.1%)
Negative	76 (24.4%)	73 (23.9%)
**Smoking**—*n* (%)		
Never-smoker	76 (24.4%)	76 (24.8%)
Smoker	143 (46.0%)	142 (46.4%)
Pack-years—median (IQR)	20 (10–40)	20 (10–40)
Pack-years unknown—*n*	20	20
Unknown	92 (29.6%)	88 (28.8 %)
**T stage**^3^—*n* (%)		
T1	43 (13.8%)	42 (13.7%)
T2	120 (38.6%)	120 (39.2%)
T3	99 (31.8%)	97 (31.7%)
T4	49 (15.8%)	47 (15.4%)
**N stage**^3^—*n* (%)		
N0	60 (19.3%)	59 (19.3%)
N1	149 (47.9%)	149 (48.7%)
N2	97 (31.2%)	94 (30.7%)
N3	5 (1.6%)	4 (1.3 %)
**Overall stage**^3^—*n* (%)		
I	117 (37.6%)	117 (38.2%)
II	91 (29.3%)	91 (29.7%)
III	50 (16.1%)	47 (15.4%)
IV	53 (17.0%)	51 (16.7%)
**Included lymph nodes/patient**—range	0–8	0–8
**Primary treatment—***n* (%)		
CCRT or CBRT	208 (66.9%)	204 (66.7%)
RT alone	28 (9.0%)	27 (8.8%)
Surgery		
Without adjuvant therapy	13 (4.2%)	13 (4.2%)
With adjuvant RT, CCRT, or CBRT	62 (19.9%)	62 (20.3%)
**PET**^4^—mean (SD)		
Slice thickness (mm)	3.40 (0.38)	3.39 (0.38)
In-plane pixel spacing (mm)	4.30 (0.91)	4.30 (0.92)
In-plane image matrix (n × n)	147.16 (58.88) × idem	147.32 (59.34) × idem
**CT**^4^—mean (SD)		
Slice thickness (mm)	3.12 (0.55)	3.10 (0.54)
In-plane pixel spacing (mm)	1.12 (0.18)	1.12 (0.18)
In-plane image matrix (n × n)	512 × 512	512 × 512

^1^ After exclusion of patients with uneventful follow-up <18 months from each respective study arm, subject counts differ in the progression-free survival (PFS) and overall survival (OS) cohorts. ^2^ The American Joint Committee on Cancer (AJCC) staging, demographics, treatment, and imaging characteristics of human papillomavirus (HPV)-associated and HPV-negative subjects are separately reported in Tables S1 and S2. ^3^ AJCC 8th edition staging manual T/N/overall stage [9]. ^4^ Values are from original images before pre-processing. CBRT = concurrent bioradiotherapy with cetuximab; CCRT = concurrent platinum-based chemoradiotherapy; IQR = interquartile range; RT = radiotherapy; SD = standard deviation; TCIA = The Cancer Imaging Archive.

## Supplementary Materials

The following are available online at https://www.mdpi.com/2072-6694/12/7/1778/s1, Figure S1: Heatmap depicting mean Harrell´s C-index ± SD in validation folds across 33 repeats of threefold stratified cross validation. Figure S2: Kaplan–Meier plots with log-rank test p-values depicting radiomics- and AJCC-based risk stratification in HPV-associated (a,b) and HPV-negative (c,d) cohorts in the OS and PFS study arms. Table S1: Patients’ characteristics: HPV-associated cancers. Table S2 Patients’ characteristics: HPV-negative cancers. Table S3: List of extracted radiomics features. Table S4: Multiple delineation-based feature stability assessment.

## Abbreviations

AJCC	American Joint Committee on Cancer
AUC	area under the receiver operating characteristic curve
CT	computed tomography
HPV	human papillomavirus
IQR	interquartile range
OPSCC	oropharyngeal squamous cell carcinoma
OS	overall survival
PET	[18F]fluorodeoxyglucose positron emission tomography
PFS	progression-free survival
RCF	random classification forest
RSF	random survival forest
SD	standard deviation
TCIA	The Cancer Imaging Archive
UICC	Union for International Cancer Control
VOI	volume of interest
