# Supplementary material for: Potential Added Value of PET/CT Radiomics for Survival Prognostication beyond AJCC 8th Edition Staging in Oropharyngeal Squamous Cell Carcinoma
Source: Cancers (Basel). 2020 Jul 3;12(7):1778. doi: 10.3390/cancers12071778 (PMC7407414; doi:10.3390/cancers12071778)
Supplement: Supplementary file 1 [file cancers-12-01778-s001.pdf]

Supplement for the original article

Potential Added Value of PET/CT Radiomics for Survival Prognostication beyond AJCC 8th Edition Staging in Oropharyngeal Squamous Cell Carcinoma

Stefan P. Haider; Tal Zeevi, MSc; Philipp Baumeister, Christoph Reichel, Kariem Sharaf, Reza Forghani, Benjamin H. Kann, Benjamin L. JudsonManju L. Prasad, Barbara Burtness, Amit Mahajan and Seyedmehdi Payabvash,

1. Supplementary Methods

1.1. Image Pre-Processing Pipeline

For normalization of PET scan voxel values, we divided each voxel's intensity by the left lentiform nucleus' maximum intensity to improve the inter-scanner and inter-institutional generalizability of PET-based quantitative metrics [1]. To ensure texture feature rotational invariance [2] and even out voxel size and slice thickness dissimilarities [3–6], we generated isotropic 3 × 3 × 3 and 2 × 2 × 2 mm PET and CT voxels, respectively, using trilinear image interpolation [7]. A re-segmentation process of CT volumes of interest (VOI) only retaining voxels within a 1–300 Hounsfield unit (HU) range was applied to restrict radiomics analysis to soft tissue densities. We generated ten image derivates per original PET or CT scan to refine radiomics analysis of specific characteristics: High and low frequency analysis was enhanced using a "coif-1" wavelet transform to generate eight decompositions per original [7,8]. Laplacian of Gaussian (LoG) filtering for edge-enhancement with "sigma" settings of 3 and 6 mm for PET, and 2 and 4 mm for CT images yielded two additional derivates per original scan [7,9]. To enable extraction of texture and first-order features [2], voxel intensities were discretized using a fixed-bin-width method [7,10] with a 2 unit width for PET and CT scans. We customized a Pyradiomics version 2.1.2 pipeline to facilitate image pre-processing [7,11].

1.2. Ancillary Study to Determine Feature Robustness

Given the variable robustness of individual radiomics features to segmentation inconsistencies, we conducted a multiple delineation-based feature pre-selection study to exclude features with low inter- and intra-observer stability from the feature set utilized in this study. Imaging data was acquired from three collections provided by a public imaging repository ("The Cancer Imaging Archive", TCIA) [12] – including (1) the "Head-Neck-PET-CT" collection from four Canadian centers [13,14]; (2) the "Head and Neck Cancer CT Atlas" collection from MD Anderson Cancer Center dataset [15,16]; and (3) the "TCGA-HNSC" collection from various institutions across the United States [17].

Subjects with (1) pre-treatment PET and non-contrast CT scans of the neck, (2) biopsy-confirmed OPSCC, and (3) known p16 or high-risk HPV status were included. Patients with (1) recurrent OPSCC, or (2) >50% of the primary tumor VOI affected by CT artifacts [18] were excluded.

A randomly sampled cohort of 50 patients from the pooled TCIA cohorts (stratified by dataset) was selected. Observer 1 segmented all primary tumors and two randomly selected metastatic nodes in each patient; and re-segmented the same set of lesions >2 months after initial review and segmentation. A second observer created a third set of segmentations. After feature extraction, two intraclass correlation coefficient (ICC) statistics were calculated for each radiomics feature: To assess inter-rater agreement, a two-way random effects, absolute agreement, single rater/measurement ICC was applied; and the two-way mixed effects, absolute agreement, single rater/measurement ICC was used to quantify intra-rater agreement [19,20]. Features with a lower 95% confidence interval bound ≥0.8 in both inter- and intra-rater assessments

were retained for further analysis. ICC metrics were separately calculated for primary tumors and the combined set of tumors and nodes. The R "psych" package [21] "ICC" function was used for ICC calculations.

Table S4 summarizes the results – feature sets exhibited similar inter- and intra-rater ICC scores. PET feature reproducibility was superior to CT in primary tumors, but inferior in the combined set of all lesions. The number and ratio of features retained for further analysis in each subset are reported in Table S4.

1.3. Dimensionality Reduction Techniques

1.3.1. HClust – Hierarchical Clustering

The R "stats" package (version 3.6.0) [22] "dist" function was used to generate a "euclidean" radiomics feature distance matrix. Next, the "stats" "hclust" function performed hierarchical clustering, applying Ward clustering with Ward's clustering criterion implemented (i.e. "ward.D2" package option) [23]. We cut the dendrogram and retained 30 clusters ("stats" "cutree" function). One "meta-feature" was extracted from each cluster by averaging all radiomics features. Clustering was performed with cross-validation training data only, and meta-feature computation was subsequently applied in all subjects.

1.3.2. None – No Feature Selection

Feature dimensionality reduction was omitted, and the random survival forest models were fit on the unreduced feature set.

1.3.3. pRF – Pearson Correlation-Based Redundancy Reduction with Random Forest Variable Importance

The R "stats" package (version 3.6.0) [22] "cor" function was configured to compute a radiomics feature correlation matrix utilizing Pearson's correlation coefficient (r) based on the cross-validation training set. To reduce pair-wise feature correlation, we excluded the feature with higher mean absolute correlation from any given feature pair with $r > 0.9$ or $r < -0.9$ ("findCorrelation" function of "caret" package) [24].

Thereafter, a random survival forest model was fit on the dimensionality-reduced cross-validation training data ("ranger" package version 0.12.1 [25]). A C-index based split rule [26] was applied to grow 1000 decision trees with the remaining function arguments kept in default. Radiomics feature variable importance scores were queried from the random forest object, and features were ranked in descending order of their respective importance score. The 30 highest-ranked features were selected for survival modelling.

1.3.4. RIDGE – RIDGE Regularized Cox Regression for Feature Selection

Ridge-regularized Cox survival regression models were trained using the cross-validation training folds ("glmnet" package version 2.0-18 [27] "cv.glmnet" function). The "lambda" parameter was automatically determined in 10-fold cross validation within the "cv.glmnet" fitting process, and each feature's regression coefficient was derived from the fit model at the "lambda" value minimizing the mean cross-validated error. Features were ranked in descending order of their respective absolute regression coefficient value, and the 30 highest-ranked features were selected for survival modelling.

2. Supplementary Tables

Table S1. Patients' Characteristics: HPV-associated Cancers.

Survival Endpoint	Progression-free Survival	Overall Survival
**Number of patients** – n	235	233
**Included lymph nodes** – n	348	341
**Events** – n (%)	51 (21.7 %)	30 (12.9 %)
**Follow-up [days]** – median (IQR)	1226 (875 – 1658)	1237 (888 – 1659)
**Data source** – n (%)		
Yale	153 (65.1 %)	153 (65.7 %)
TCIA	82 (34.9 %)	80 (34.3 %)
**Sex** – n (%)		
male	192 (81.7 %)	190 (81.5 %)
female	43 (18.3 %)	43 (18.5 %)
**Age [years]** – mean (SD)	60.26 (8.85)	60.23 (8.88)
**HPV status** – n (%)		
positive	235 (100 %)	233 (100 %)
negative	0 (0 %)	0 (0 %)
**Smoking** – n (%)		
never-smoker	68 (28.9 %)	68 (29.2 %)
smoker	98 (41.7 %)	98 (42.1 %)
pack-years – median (IQR)	18 (8.75-30)	18 (8.75-30)
pack-years unknown – n	15	15
unknown	69 (29.4 %)	67 (28.8 %)
**T stage** [1] – n (%)		
T1	34 (14.5 %)	33 (14.2 %)
T2	101 (43.0 %)	101 (43.3 %)
T3	72 (30.6 %)	72 (30.9 %)
T4	28 (11.9 %)	27 (11.6 %)
**N stage** [1] – n (%)		
N0	44 (18.7 %)	44 (18.9 %)
N1	133(56.6 %)	133 (57.1 %)
N2	53 (22.6 %)	52 (22.3 %)
N3	5 (2.1 %)	4 (1.7 %)
**Overall stage** [1] – n (%)		
I	113 (48.1 %)	113 (48.5 %)
II	89 (37.9 %)	89 (38.2 %)
III	33 (14.0 %)	31 (13.3 %)
**Included lymph nodes / patient** – range	0 – 8	0 – 8
**Primary treatment** – n (%)		
CCRT or CBRT	150 (63.8 %)	148 (63.5 %)
RT alone	22 (9.4 %)	22 (9.4 %)
surgery		
without adjuvant therapy	10 (4.3 %)	10 (4.3 %)
with adjuvant RT, CCRT or CBRT	53 (22.6 %)	53 (22.7 %)
**PET** [2] – mean (SD)		
slice thickness [mm]	3.40 (0.40)	3.40 (0.40)
in-plane pixel spacing [mm]	4.28 (0.94)	4.28 (0.94)
in-plane image matrix [n x n]	149.96 (65.85) x idem	150.08 (66.12) x idem
**CT** [2] – mean (SD)		
slice thickness [mm]	3.08 (0.57)	3.08 (0.58)
in-plane pixel spacing [mm]	1.12 (0.19)	1.12 (0.19)
in-plane image matrix [n x n]	512 x 512	512 x 512

[1] AJCC 8th edition staging manual T/N/overall stage [28]; [2] Values are from original images before pre-processing; CBRT = concurrent bioradiotherapy with cetuximab; CCRT = concurrent platinum-based chemoradiotherapy; IQR = interquartile range; RT = radiotherapy; SD = standard deviation; TCIA = The Cancer Imaging Archive

Table S2. Patients' Characteristics: HPV-negative Cancers.

Survival Endpoint	Progression-free Survival	Overall Survival
**Number of patients** – n	76	73
**Included lymph nodes** – n	127	121
**Events** – n (%)	43 (56.6 %)	28 (38.4 %)
**Follow-up [days]** – median (IQR)	945.5 (667.25–1479)	979 (694–1527)
**Data source** – n (%)		
Yale	48 (63.2 %)	47 (64.4 %)
TCIA	28 (36.8 %)	26 (35.6 %)
**Sex** – n (%)		
male	61 (80.3 %)	59 (80.8 %)
female	15 (19.7 %)	14 (19.2 %)
**Age [years]** – mean (SD)	61.68 (10.35)	61.79 (10.45)
**HPV status** – n (%)		
positive	0 (0 %)	0 (0 %)
negative	76 (100 %)	73 (100 %)
**Smoking** – n (%)		
never-smoker	8 (10.5 %)	8 (11.0 %)
smoker	45 (59.2 %)	44 (60.3 %)
pack-years – median (IQR)	30 (14.38-50)	30 (13.75-45)
pack-years unknown – n	5	5
unknown	23 (30.3 %)	21 (28.8 %)
**T stage** [1] – n (%)		
T1	9 (11.8 %)	9 (12.3 %)
T2	19 (25.0 %)	19 (26.0 %)
T3	27 (35.5 %)	25 (34.2 %)
T4	21 (27.6 %)	20 (27.4 %)
**N stage** [1] – n (%)		
N0	16 (21.1 %)	15 (20.5 %)
N1	16 (21.1 %)	16 (21.9 %)
N2	44 (57.9 %)	42 (57.5 %)
N3	0 (0 %)	0 (0 %)
**Overall stage** [1] – n (%)		
I	4 (5.3 %)	4 (5.5 %)
II	2 (2.6 %)	2 (2.7 %)
III	17 (22.4 %)	16 (21.9 %)
IV	53 (69.7 %)	51 (69.9 %)
**Included lymph nodes / patient** – range	0 – 8	0 – 8
**Primary treatment** – n (%)		
CCRT or CBRT	58 (76.3 %)	56 (76.7 %)
RT alone	6 (7.9 %)	5 (6.8 %)
surgery		
without adjuvant therapy	3 (3.9 %)	3 (4.1 %)
with adjuvant RT, CCRT or CBRT	9 (11.8 %)	9 (12.3 %)
**PET** [2] – mean (SD)		
slice thickness [mm]	3.39 (0.28)	3.38 (0.27)
in-plane pixel spacing [mm]	4.36 (0.85)	4.36 (0.86)
in-plane image matrix [n x n]	138.53 (26.39) x idem	138.52 (26.89) x idem
**CT** [2] – mean (SD)		
slice thickness [mm]	3.23 (0.48)	3.18 (0.38)
in-plane pixel spacing [mm]	1.11 (0.18)	1.10 (0.18)
in-plane image matrix [n x n]	512 x 512	512 x 512

[1] AJCC 8th edition staging manual T/N/overall stage [28]; [2] Values are from original images before pre-processing; CBRT = concurrent bioradiotherapy with cetuximab; CCRT = concurrent platinum-based chemoradiotherapy; IQR = interquartile range; RT = radiotherapy; SD = standard deviation; TCIA = The Cancer Imaging Archive

Table S3. List of Extracted Radiomics Features.

Feature Family		Feature Name
**First-order**	1	10th percentile
	2	90th percentile
	3	Energy
	4	Entropy
	5	Interquartile Range
	6	Kurtosis
	7	Maximum
	8	Mean
	9	Mean Absolute Deviation
	10	Median
	11	Minimum
	12	Range
	13	Robust Mean Absolute Deviation
	14	Root Mean Squared
	15	Skewness
	16	Total Energy
	17	Uniformity
	18	Variance
**Shape**	1	Elongation
	2	Flatness
	3	Least Axis Length
	4	Major Axis Length
	5	Maximum 2D Diameter (Column)
	6	Maximum 2D Diameter (Row)
	7	Maximum 2D Diameter (Slice)
	8	Maximum 3D Diameter
	9	Mesh Volume
	10	Minor Axis Length
	11	Sphericity
	12	Surface Area
	13	Surface Area to Volume Ratio
	14	Voxel Volume
**Texture - Gray Level Cooccurrence Matrix Features**	1	Autocorrelation
	2	Cluster Prominence
	3	Cluster Shade
	4	Cluster Tendency
	5	Contrast
	6	Correlation
	7	Difference Average
	8	Difference Entropy
	9	Difference Variance
	10	Informational Measure of Correlation 1

Feature Family		Feature Name
	11	Informational Measure of Correlation 2
	12	Inverse Difference
	13	Inverse Difference Moment
	14	Inverse Difference Moment Normalized
	15	Inverse Difference Normalized
	16	Inverse Variance
	17	Joint Average
	18	Joint Energy
	19	Joint Entropy
	20	Maximal Correlation Coefficient
	21	Maximum Probability
	22	Sum Average
	23	Sum Entropy
	24	Sum of Squares
**Texture - Gray Level Size Zone Matrix Features**	1	Gray Level Non-Uniformity
	2	Gray Level Non-Uniformity Normalized
	3	Gray Level Variance
	4	High Gray Level Zone Emphasis
	5	Large Area Emphasis
	6	Large Area High Gray Level Emphasis
	7	Large Area Low Gray Level Emphasis
	8	Low Gray Level Zone Emphasis
	9	Size Zone Non-Uniformity
	10	Size Zone Non-Uniformity Normalized
	11	Small Area Emphasis
	12	Small Area High Gray Level Emphasis
	13	Small Area Low Gray Level Emphasis
	14	Zone Entropy
	15	Zone Percentage
	16	Zone Variance
**Texture - Gray Level Run Length Matrix Features**	1	Gray Level Non-Uniformity
	2	Gray Level Non-Uniformity Normalized
	3	Gray Level Variance
	4	High Gray Level Run Emphasis
	5	Long Run Emphasis

Feature Family		Feature Name
	6	Long Run High Gray Level Emphasis
	7	Long Run Low Gray Level Emphasis
	8	Low Gray Level Run Emphasis
	9	Run Entropy
	10	Run Length Non-Uniformity
	11	Run Length Non-Uniformity Normalized
	12	Run Percentage
	13	Run Variance
	14	Short Run Emphasis
	15	Short Run High Gray Level Emphasis
	16	Short Run Low Gray Level Emphasis
**Texture - Neighboring Gray Tone Difference Matrix Features**	1	Busyness
	2	Coarseness
	3	Complexity
	4	Contrast
	5	Strength
**Texture - Gray Level Dependence Matrix Features**	1	Dependence Entropy
	2	Dependence Non-Uniformity
	3	Dependence Non-Uniformity Normalized
	4	Dependence Variance
	5	Gray Level Non-Uniformity
	6	Gray Level Variance
	7	High Gray Level Emphasis
	8	Large Dependence Emphasis
	9	Large Dependence High Gray Level Emphasis
	10	Large Dependence Low Gray Level Emphasis
	11	Low Gray Level Emphasis
	12	Small Dependence Emphasis
	13	Small Dependence High Gray Level Emphasis
	14	Small Dependence Low Gray Level Emphasis

Complete list of Pyradiomics [11] features used in this study. Exact feature definitions are provided in ref. [7]

Table S4. Multiple Delineation-based Feature Stability Assessment.

Radiomics Source VOI	Number of lesions (n)	Mean inter-Rater ICC (SD)	Mean Intra-Rater ICC (SD)	Number of Retained Features (%)
**Primary tumors**	50	**PET**: 0.92 (0.12)<br>**CT**: 0.86 (0.16)	**PET**: 0.91 (0.11)<br>**CT**: 0.89 (0.13)	**PET**: 751 (72.4 %)<br>**CT**: 586 (54.7 %)
**Primary tumors and lymph nodes**	50 (tumor lesions)<br>65 (lymph nodes)	**PET**: 0.88 (0.15)<br>**CT**: 0.91 (0.13)	**PET**: 0.87 (0.16)<br>**CT**: 0.93 (0.11)	**PET**: 651 (62.8 %)<br>**CT**: 854 (82.4 %)

Based on three VOI sets created by two observers, inter- and intra-rater ICC were calculated for each feature in primary tumor lesions and a combined set of tumor and lymph node VOI. The mean (SD) ICC in PET and CT feature subsets is reported as well as the number (%) of features retained for further analysis (lower 95% confidence interval bound of inter- and intra-rater ICC ≥0.8).

3. Supplementary Figures

a) HPV-pos — Selected models (boxed; shown in bold below)

VOI	Modality	Method	PFS Combined	PFS Radiomics	PFS AJCC	OS Combined	OS Radiomics	OS AJCC
**VOI:** Consensus of tumor and nodes	PET/CT	HClust	0.58±0.05	0.56±0.05	0.54±0.06	0.56±0.06	0.58±0.07	0.55±0.08
		none	**0.62±0.05**	**0.62±0.05**		0.60±0.06	0.61±0.06	
		pRF	0.59±0.06	0.59±0.06		0.56±0.07	0.57±0.08	
		RIDGE	0.58±0.06	0.57±0.06		0.56±0.09	0.57±0.09	
	PET	HClust	0.59±0.05	0.58±0.06	0.54±0.06	0.61±0.07	0.61±0.08	0.55±0.08
		none	0.61±0.06	0.61±0.06		**0.63±0.08**	**0.63±0.08**	
		pRF	0.58±0.07	0.57±0.07		0.58±0.08	0.58±0.08	
		RIDGE	0.56±0.06	0.56±0.06		0.59±0.08	0.60±0.08	
	CT	HClust	0.54±0.05	0.52±0.06	0.54±0.06	0.50±0.07	0.54±0.08	0.55±0.08
		none	0.56±0.05	0.56±0.06		0.53±0.07	0.53±0.08	
		pRF	0.56±0.06	0.55±0.06		0.51±0.08	0.53±0.09	
		RIDGE	0.55±0.06	0.54±0.06		0.50±0.09	0.51±0.10	
**VOI:** Primary tumor	PET/CT	HClust	0.57±0.06	0.58±0.07	0.54±0.06	0.57±0.08	0.60±0.08	0.55±0.08
		none	0.59±0.06	0.58±0.06		0.58±0.07	0.59±0.07	
		pRF	0.58±0.06	0.58±0.06		0.56±0.07	0.57±0.08	
		RIDGE	0.56±0.05	0.55±0.06		0.56±0.10	0.58±0.11	
	PET	HClust	0.58±0.06	0.58±0.06	0.54±0.06	0.57±0.08	0.59±0.08	0.55±0.08
		none	0.60±0.06	0.60±0.06		0.58±0.08	0.59±0.08	
		pRF	0.59±0.06	0.59±0.06		0.57±0.08	0.59±0.08	
		RIDGE	0.58±0.06	0.57±0.06		0.58±0.10	0.60±0.10	
	CT	HClust	0.56±0.05	0.57±0.06	0.54±0.06	0.54±0.07	0.58±0.07	0.55±0.08
		none	0.54±0.06	0.54±0.06		0.57±0.07	0.58±0.07	
		pRF	0.55±0.05	0.54±0.06		0.53±0.08	0.56±0.09	
		RIDGE	0.55±0.05	0.55±0.06		0.52±0.09	0.54±0.09	

Harrell´s C-index: 0.65 / 0.55 / 0.45

b) HPV-neg			PFS Combined	PFS Radiomics	PFS AJCC	OS Combined	OS Radiomics	OS AJCC
VOI: Consensus of tumor and nodes	PET/CT	HClust	0.53±0.08	0.54±0.08	0.50±0.06	0.60±0.08	0.60±0.08	0.50±0.08
		none	0.55±0.08	0.55±0.08		0.58±0.08	0.58±0.08	
		pRF	0.53±0.07	0.55±0.08		0.54±0.10	0.54±0.10	
		RIDGE	0.51±0.08	0.52±0.07		0.54±0.10	0.54±0.11	
	PET	HClust	0.56±0.07	0.52±0.07	0.50±0.06	0.59±0.08	0.58±0.08	0.50±0.08
		none	0.54±0.07	0.54±0.07		0.59±0.09	0.59±0.09	
		pRF	0.54±0.08	0.54±0.08		0.58±0.09	0.58±0.09	
		RIDGE	0.53±0.08	0.52±0.09		0.59±0.10	0.58±0.11	
	CT	HClust	0.46±0.08	0.51±0.07	0.50±0.06	0.54±0.08	0.56±0.10	0.50±0.08
		none	0.52±0.07	0.53±0.07		0.54±0.08	0.54±0.09	
		pRF	0.50±0.07	0.52±0.07		0.52±0.09	0.52±0.09	
		RIDGE	0.49±0.07	0.51±0.07		0.50±0.09	0.50±0.09	
VOI: Primary tumor	PET/CT	HClust	0.53±0.06	0.53±0.06	0.50±0.06	0.58±0.08	0.58±0.08	0.50±0.08
		none	0.53±0.07	0.53±0.07		0.59±0.08	0.59±0.08	
		pRF	0.51±0.07	0.52±0.07		0.57±0.08	0.57±0.08	
		RIDGE	0.52±0.07	0.52±0.08		0.53±0.09	0.54±0.09	
	PET	HClust	0.54±0.07	0.52±0.08	0.50±0.06	0.57±0.08	0.57±0.08	0.50±0.08
		none	0.53±0.08	0.52±0.08		0.55±0.07	0.55±0.07	
		pRF	0.53±0.08	0.53±0.09		0.56±0.08	0.56±0.09	
		RIDGE	0.53±0.08	0.51±0.08		0.54±0.08	0.55±0.08	
	CT	HClust	0.51±0.06	0.55±0.07	0.50±0.06	0.58±0.09	0.59±0.09	0.50±0.08
		none	0.53±0.06	0.54±0.07		0.60±0.09	0.60±0.09	
		pRF	0.50±0.07	0.51±0.08		0.57±0.09	0.58±0.09	
		RIDGE	0.52±0.07	0.54±0.08		0.54±0.09	0.55±0.09	

Selected models

Harrell´s C-index: 0.65 0.55 0.45

Figure S1. Heatmap depicting mean Harrell´s C-index ± SD in validation folds across 33 repeats of 3-fold stratified cross validation.

AJCC = AJCC model; Combined = combined model; HClust = hierarchical clustering; none = no dimensionality reduction applied; OS = overall survival; PFS = progression-free survival; pRF = Pearson correlation-based redundancy reduction with random survival forest variable importance; Radiomics = radiomics model; RIDGE = Cox regression with RIDGE regularization adapted for feature selection; SD = standard deviation.

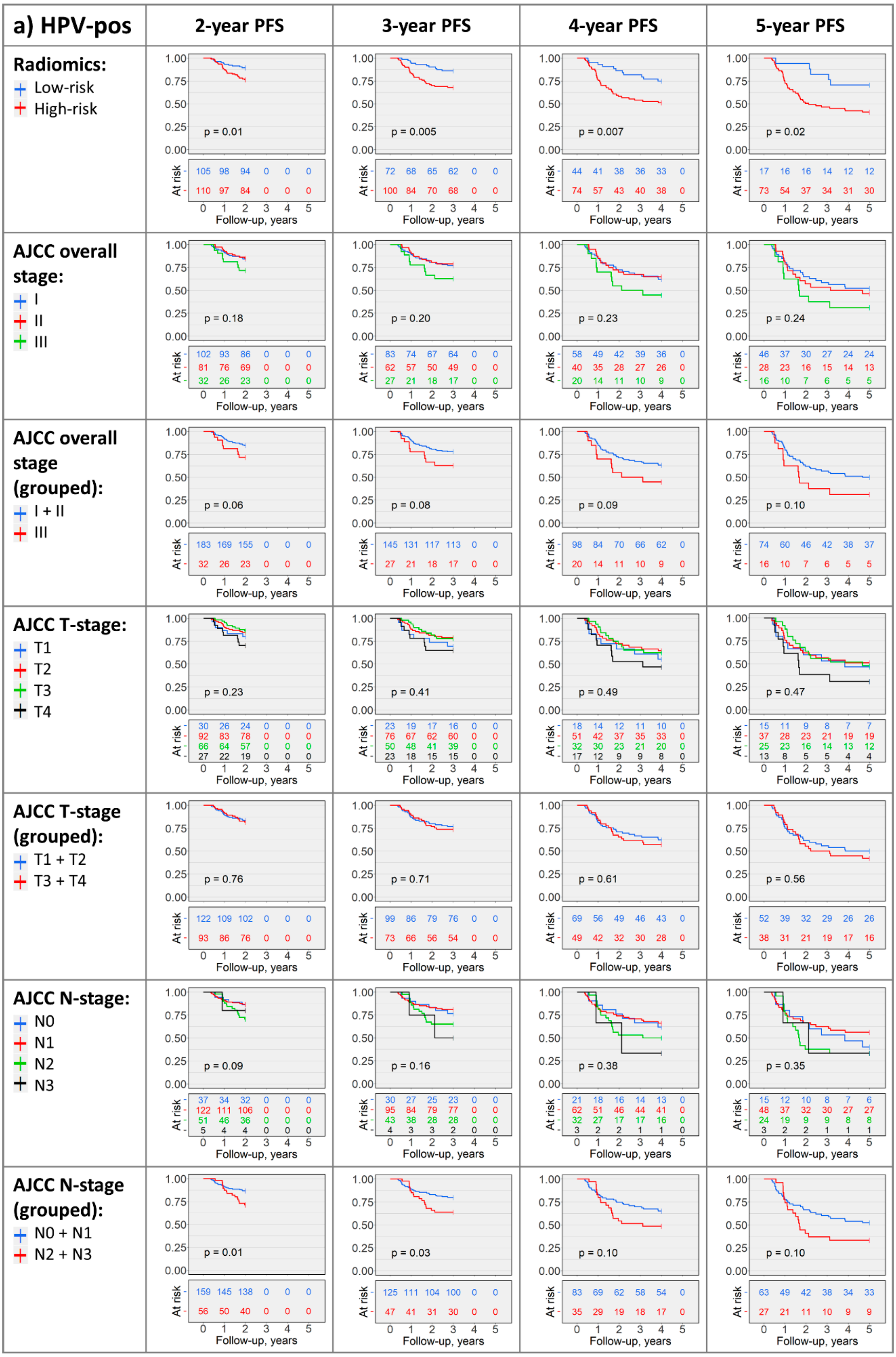
a) HPV-pos
2-year PFS
3-year PFS
4-year PFS
5-year PFS
Radiomics:
Low-risk
High-risk
p = 0.01
p = 0.005
p = 0.007
p = 0.02
AJCC overall stage:
I
II
III
p = 0.18
p = 0.20
p = 0.23
p = 0.24
AJCC overall stage (grouped):
I + II
III
p = 0.06
p = 0.08
p = 0.09
p = 0.10
AJCC T-stage:
T1
T2
T3
T4
p = 0.23
p = 0.41
p = 0.49
p = 0.47
AJCC T-stage (grouped):
T1 + T2
T3 + T4
p = 0.76
p = 0.71
p = 0.61
p = 0.56
AJCC N-stage:
N0
N1
N2
N3
p = 0.09
p = 0.16
p = 0.38
p = 0.35
AJCC N-stage (grouped):
N0 + N1
N2 + N3
p = 0.01
p = 0.03
p = 0.10
p = 0.10
At risk
Follow-up, years

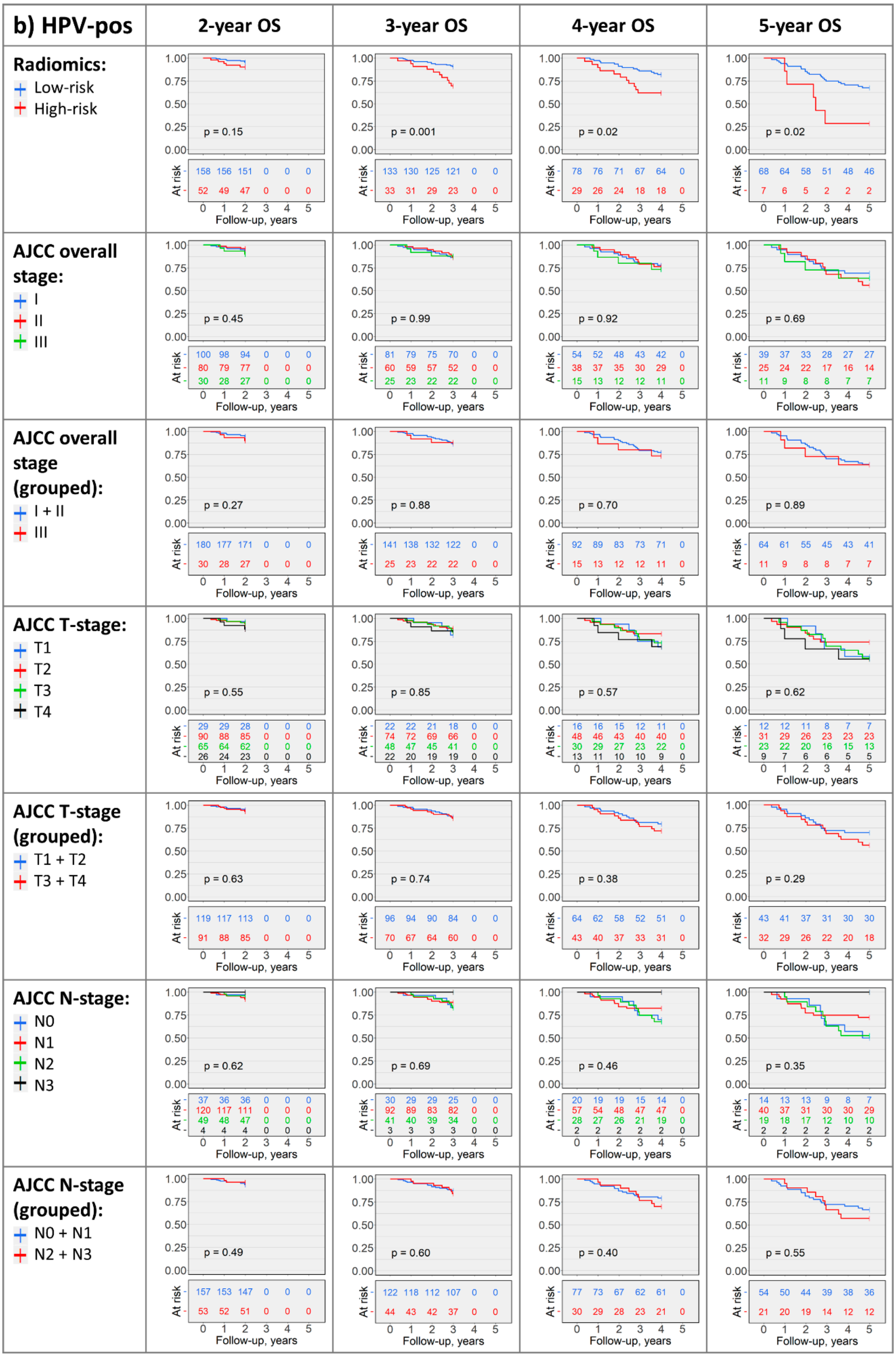

b) HPV-pos
2-year OS
3-year OS
4-year OS
5-year OS
Radiomics:
Low-risk
High-risk
p = 0.15
p = 0.001
p = 0.02
p = 0.02
AJCC overall stage:
I
II
III
p = 0.45
p = 0.99
p = 0.92
p = 0.69
AJCC overall stage (grouped):
I + II
III
p = 0.27
p = 0.88
p = 0.70
p = 0.89
AJCC T-stage:
T1
T2
T3
T4
p = 0.55
p = 0.85
p = 0.57
p = 0.62
AJCC T-stage (grouped):
T1 + T2
T3 + T4
p = 0.63
p = 0.74
p = 0.38
p = 0.29
AJCC N-stage:
N0
N1
N2
N3
p = 0.62
p = 0.69
p = 0.46
p = 0.35
AJCC N-stage (grouped):
N0 + N1
N2 + N3
p = 0.49
p = 0.60
p = 0.40
p = 0.55
At risk
Follow-up, years

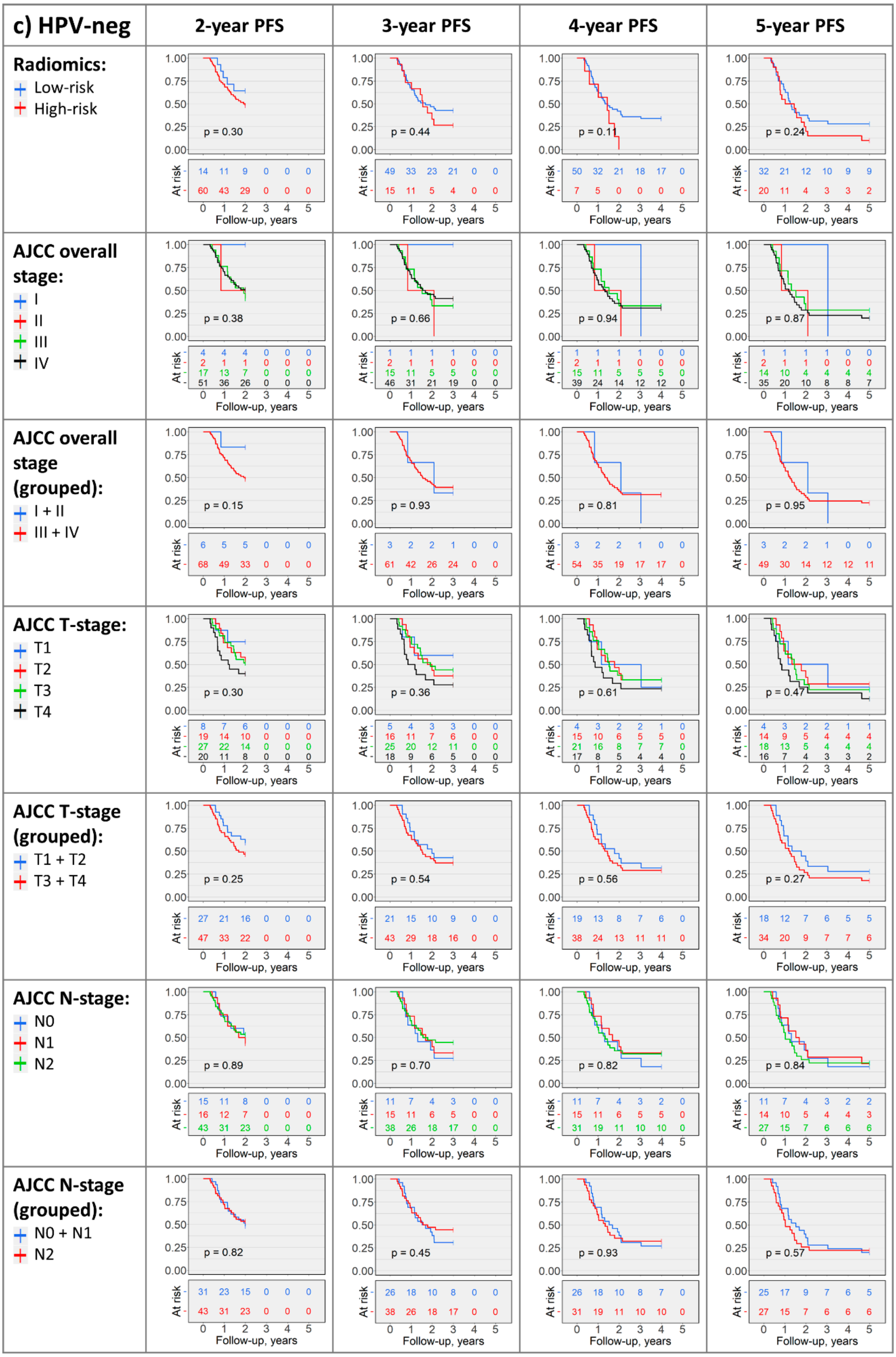
c) HPV-neg
2-year PFS
3-year PFS
4-year PFS
5-year PFS
Radiomics:
Low-risk
High-risk
p = 0.30
p = 0.44
p = 0.11
p = 0.24
AJCC overall stage:
I
II
III
IV
p = 0.38
p = 0.66
p = 0.94
p = 0.87
AJCC overall stage (grouped):
I + II
III + IV
p = 0.15
p = 0.93
p = 0.81
p = 0.95
AJCC T-stage:
T1
T2
T3
T4
p = 0.30
p = 0.36
p = 0.61
p = 0.47
AJCC T-stage (grouped):
T1 + T2
T3 + T4
p = 0.25
p = 0.54
p = 0.56
p = 0.27
AJCC N-stage:
N0
N1
N2
p = 0.89
p = 0.70
p = 0.82
p = 0.84
AJCC N-stage (grouped):
N0 + N1
N2
p = 0.82
p = 0.45
p = 0.93
p = 0.57
At risk
Follow-up, years

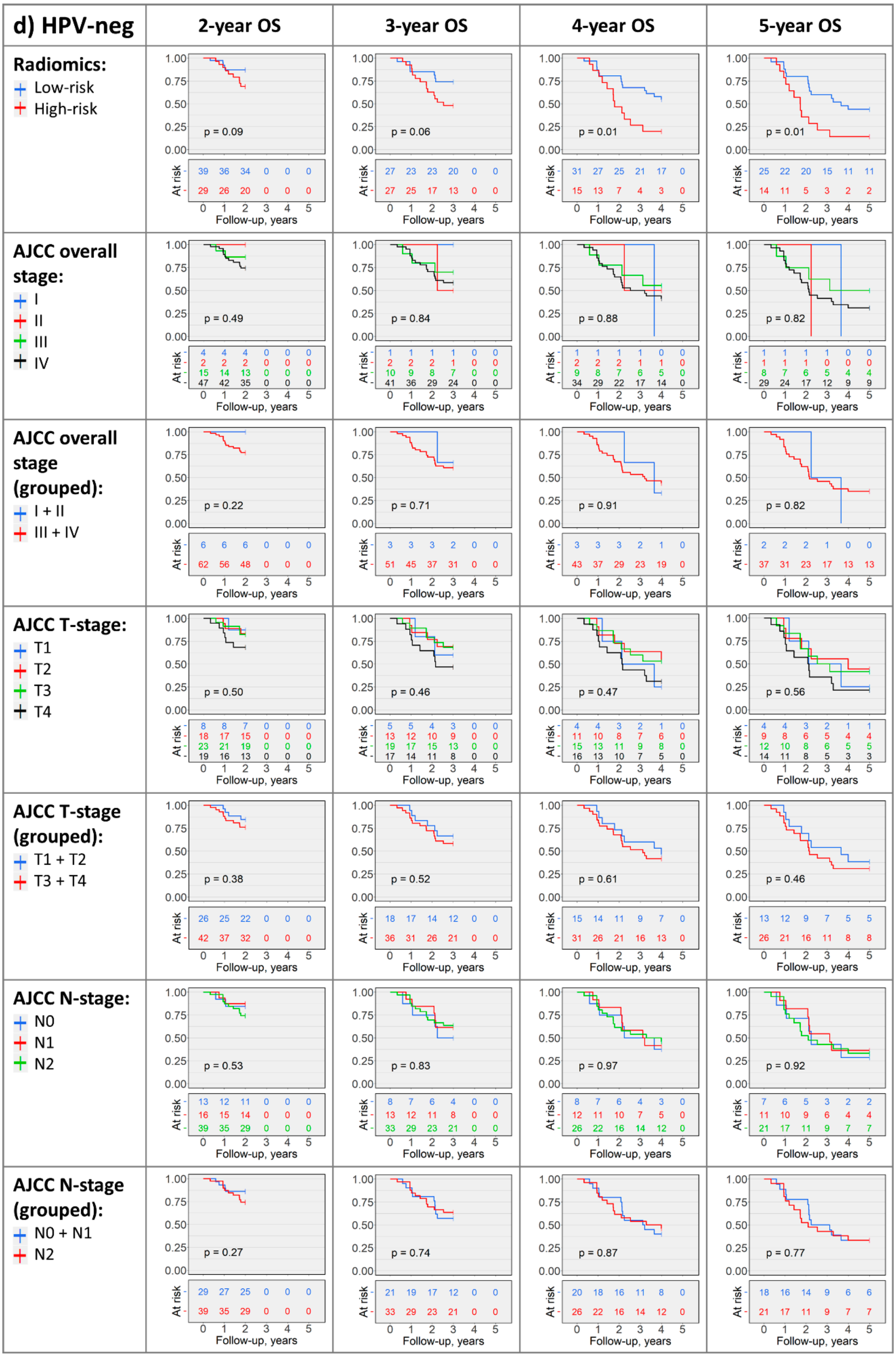

Figure S2. Kaplan-Meier plots with log-rank test p-values depicting radiomics- and AJCC-based risk stratification in HPV-associated (**a,b**) and HPV-negative (**c,d**) cohorts in the OS and PFS study arms.

OS = overall survival; PFS = progression-free survival.

References

1. Britz-Cunningham, S.H.; Millstine, J.W.; Gerbaudo, V.H. Improved discrimination of benign and malignant lesions on FDG PET/CT, using comparative activity ratios to brain, basal ganglia, or cerebellum. *Clin. Nucl. Med.* **2008**, *33*, 681–687, doi:10.1097/RLU.0b013e318184b435.
2. Zwanenburg, A.; Leger, S.; Vallières, M.; Löck, S. *Image biomarker standardisation initiative*. In arXiv e-prints, 2016.
3. Traverso, A.; Wee, L.; Dekker, A.; Gillies, R. Repeatability and Reproducibility of Radiomic Features: A Systematic Review. *Int. J. Radiat. Oncol. Biol. Phys.* **2018**, *102*, 1143–1158, doi:10.1016/j.ijrobp.2018.05.053.
4. Shafiq-Ul-Hassan, M.; Zhang, G.G.; Latifi, K.; Ullah, G.; Hunt, D.C.; Balagurunathan, Y.; Abdalah, M.A.; Schabath, M.B.; Goldgof, D.G.; Mackin, D.; et al. Intrinsic dependencies of CT radiomic features on voxel size and number of gray levels. *Med. Phys.* **2017**, *44*, 1050–1062, doi:10.1002/mp.12123.
5. Zhao, B.; Tan, Y.; Tsai, W.Y.; Qi, J.; Xie, C.; Lu, L.; Schwartz, L.H. Reproducibility of radiomics for deciphering tumor phenotype with imaging. *Sci. Rep.* **2016**, *6*, 23428, doi:10.1038/srep23428.
6. Larue, R.T.; Defraene, G.; De Ruysscher, D.; Lambin, P.; van Elmpt, W. Quantitative radiomics studies for tissue characterization: A review of technology and methodological procedures. *Br. J. Radiol.* **2017**, *90*, 20160665, doi:10.1259/bjr.20160665.
7. Pyradiomics-community. Pyradiomics Documentation Release 2.1.2. Availabe online: https://readthedocs.org/projects/pyradiomics/downloads/pdf/2.1.2/ (accessed on December 15th).
8. Aerts, H.J.; Velazquez, E.R.; Leijenaar, R.T.; Parmar, C.; Grossmann, P.; Carvalho, S.; Bussink, J.; Monshouwer, R.; Haibe-Kains, B.; Rietveld, D.; et al. Decoding tumour phenotype by noninvasive imaging using a quantitative radiomics approach. *Nat. Commun.* **2014**, *5*, 4006, doi:10.1038/ncomms5006.
9. Davnall, F.; Yip, C.S.; Ljungqvist, G.; Selmi, M.; Ng, F.; Sanghera, B.; Ganeshan, B.; Miles, K.A.; Cook, G.J.; Goh, V. Assessment of tumor heterogeneity: An emerging imaging tool for clinical practice? *Insights Imaging* **2012**, *3*, 573–589, doi:10.1007/s13244-012-0196-6.
10. Leijenaar, R.T.; Nalbantov, G.; Carvalho, S.; van Elmpt, W.J.; Troost, E.G.; Boellaard, R.; Aerts, H.J.; Gillies, R.J.; Lambin, P. The effect of SUV discretization in quantitative FDG-PET Radiomics: The need for standardized methodology in tumor texture analysis. *Sci. Rep.* **2015**, *5*, 11075, doi:10.1038/srep11075.
11. van Griethuysen, J.J.M.; Fedorov, A.; Parmar, C.; Hosny, A.; Aucoin, N.; Narayan, V.; Beets-Tan, R.G.H.; Fillion-Robin, J.C.; Pieper, S.; Aerts, H. Computational Radiomics System to Decode the Radiographic Phenotype. *Cancer Res.* **2017**, *77*, e104–e107, doi:10.1158/0008-5472.CAN-17-0339.
12. Clark, K.; Vendt, B.; Smith, K.; Freymann, J.; Kirby, J.; Koppel, P.; Moore, S.; Phillips, S.; Maffitt, D.; Pringle, M.; et al. The Cancer Imaging Archive (TCIA): Maintaining and operating a public information repository. *J. Digit. Imaging* **2013**, *26*, 1045–1057, doi:10.1007/s10278-013-9622-7.
13. Vallières, M.; Kay-Rivest, E.; Perrin, L.J.; Liem, X.; Furstoss, C.; Khaouam, N.; Nguyen-Tan, P.F.; Wang, C.; Sultanem, K. Data from Head-Neck-PET-CT. The Cancer Imaging Archive, **2017**; 10.7937/K9/TCIA.2017.8oje5q00.
14. Vallieres, M.; Kay-Rivest, E.; Perrin, L.J.; Liem, X.; Furstoss, C.; Aerts, H.; Khaouam, N.; Nguyen-Tan, P.F.; Wang, C.S.; Sultanem, K.; et al. Radiomics strategies for risk assessment of tumour failure in head-and-neck cancer. *Sci. Rep.* **2017**, *7*, 10117, doi:10.1038/s41598-017-10371-5.
15. Grossberg, A.; Mohamed, A.; Elhalawani, H.; Bennett, W.; Smith, K.; Nolan, T.; Chamchod, S.; Kanto, r.M.; Browne, T.; Hutcheson, K.; et al. Data from Head and Neck Cancer CT Atlas. *The Cancer Imaging Archive*, **2017**; 10.7937/K9/TCIA.2017.umz8dv6s.
16. Grossberg, A.J.; Mohamed, A.S.R.; Elhalawani, H.; Bennett, W.C.; Smith, K.E.; Nolan, T.S.; Williams, B.; Chamchod, S.; Heukelom, J.; Kantor, M.E.; et al. Imaging and clinical data archive for head and neck squamous cell carcinoma patients treated with radiotherapy. *Sci. Data* **2018**, *5*, 180173, doi:10.1038/sdata.2018.173.
17. Zuley, M.L.; Jarosz, R.; Kirk, S.; Lee, Y.; Colen, R.; Garcia, K.; Aredes, N.D. Radiology Data from The Cancer Genome Atlas Head-Neck Squamous Cell Carcinoma [TCGA-HNSC] collection. *The Cancer Imaging Archive*, **2016**; 10.7937/K9/TCIA.2016.LXKQ47MS.
18. Ger, R.B.; Craft, D.F.; Mackin, D.S.; Zhou, S.; Layman, R.R.; Jones, A.K.; Elhalawani, H.; Fuller, C.D.; Howell, R.M.; Li, H.; et al. Practical guidelines for handling head and neck computed tomography artifacts for quantitative image analysis. *Comput. Med. Imaging Graph.* **2018**, *69*, 134–139, doi:10.1016/j.compmedimag.2018.09.002.

19. McGraw, K.O.; Wong, S.P. Forming inferences about some intraclass correlation coefficients. *Psychological Methods* **1996**, *1*, 30–46, doi:10.1037/1082-989X.1.1.30.
20. Koo, T.K.; Li, M.Y. A Guideline of Selecting and Reporting Intraclass Correlation Coefficients for Reliability Research. *J. Chiropr. Med.* **2016**, *15*, 155–163, doi:10.1016/j.jcm.2016.02.012.
21. Revelle, W. *psych: Procedures for Psychological, Psychometric, and Personality Research*, Version 1.8.12; Northwestern University, Evanston, Illinois, USA, 2018.
22. R Development Core Team *R: A language and environment for statistical computing*, R Foundation for Statistical Computing: Vienna, Austria, 2019.
23. Murtagh, F.; Legendre, P. Ward's Hierarchical Agglomerative Clustering Method: Which Algorithms Implement Ward's Criterion? *Journal of Classification* **2014**, *31*, 274–295, doi:10.1007/s00357-014-9161-z.
24. Kuhn, M.; Contributions from Wing, J.; Weston, S.; Williams, A.; Keefer, C.; Engelhardt, A.; Cooper, T.; Mayer, Z.; Kenkel, B.; Team, t.R.C.; et al. *caret: Classification and Regression Training*, 6.0–84; **2019**.
25. Wright, M.N.; Ziegler, A. ranger: A Fast Implementation of Random Forests for High Dimensional Data in C plus plus and R. *J. Stat. Softw.* **2017**, *77*, 1–17, doi:10.18637/jss.v077.i01.
26. Schmid, M.; Wright, M.; Ziegler, A. *On the use of Harrell's C for clinical risk prediction via random survival forest*s. In arXiv e-prints, 2015.
27. Friedman, J.H.; Hastie, T.; Tibshirani, R. Regularization Paths for Generalized Linear Models via Coordinate Descent. *2010* **2010**, *33*, 22, doi:10.18637/jss.v033.i01.
28. Amin, M., Edge, S., Greene, F., Byrd, D., Brookland, R., Washington, M., Gershenwald, J., Compton, C., Hess, K.e.a., Eds. *AJCC Cancer Staging Manual (8th edition)*; Springer International Publishing: 2017.
